# The decreased connectivity in middle temporal gyrus can be used as a potential neuroimaging biomarker for left temporal lobe epilepsy

**DOI:** 10.3389/fpsyt.2022.972939

**Published:** 2022-08-10

**Authors:** Jinlong Wu, Jun Wu, Ruimin Guo, Linkang Chu, Jun Li, Sheng Zhang, Hongwei Ren

**Affiliations:** ^1^Department of Imaging Center, Tianyou Hospital Affiliated to Wuhan University of Science and Technology, Wuhan, China; ^2^Key Laboratory of Occupational Hazards and Identification, Wuhan University of Science and Technology, Wuhan, China; ^3^Department of Neurosurgery, The Central Hospital of Wuhan, Tongji Medical College, Huazhong University of Science and Technology, Wuhan, China; ^4^Liyuan Hospital of Tongji Medical College, Huazhong University of Science and Technology, Wuhan, China

**Keywords:** left temporal lobe epilepsy, voxel-mirrored homotopic connectivity, rs-fMRI, support vector machine, neuroimaging biomarker

## Abstract

**Objective:**

We aimed to explore voxel-mirrored homotopic connectivity (VMHC) abnormalities between the two brain hemispheres in left temporal lobe epilepsy (lTLE) patients and to determine whether these alterations could be leveraged to guide lTLE diagnosis.

**Materials and methods:**

Fifty-eight lTLE patients and sixty healthy controls (HCs) matched in age, sex, and education level were recruited to receive resting state functional magnetic resonance imaging (rs-fMRI) scan. Then VHMC analyses of bilateral brain regions were conducted based on the results of these rs-fMRI scans. The resultant imaging data were further analyzed using support vector machine (SVM) methods.

**Results:**

Compared to HCs, patients with lTLE exhibited decreased VMHC values in the bilateral middle temporal gyrus (MTG) and middle cingulum gyrus (MCG), while no brain regions in these patients exhibited increased VMHC values. SVM analyses revealed the diagnostic accuracy of reduced bilateral MTG VMHC values to be 75.42% (89/118) when differentiating between lTLE patients and HCs, with respective sensitivity and specificity values of 74.14% (43/58) and 76.67% (46/60).

**Conclusion:**

Patients with lTLE exhibit abnormal VMHC values corresponding to the impairment of functional coordination between homotopic regions of the brain. These altered MTG VMHC values may also offer value as a robust neuroimaging biomarker that can guide lTLE patient diagnosis.

## Introduction

Epilepsy is a common neurological disorder that causes affected patients to experience altered brain activity and recurrent seizures (1). An estimated 50 million individuals worldwide are thought to suffer from epilepsy, experiencing a range of psychiatric and psychosocial comorbidities in addition to the physical challenges and seizures that are inherent to this disease state (2, 3). TLE is among the most prevalent subtypes of partial epilepsy. While some left temporal lobe epilepsy (lTLE) patients can attain significant from antiepileptic drug (AED) treatment, others fail to achieve remission (4, 5). Findings in lTLE patients often include impaired speech activity that may coincide with damage to the left hippocampus, lateral white matter, and lateral temporal cortex regions of the brain (6, 7). Recent work suggests that rather than merely arising as a consequence of localized neurological abnormalities, epilepsy may represent a form of network disorder (8). Accordingly, the majority of patients with lTLE experience varying types of cognitive dysfunction including altered attention, consciousness, memory, or behavior after experiencing recurrent seizures (8, 9). Efforts to diagnose lTLE and other forms of epilepsy are currently based on a combination of medical history and electroencephalogram (EEG) analyses (10). While EEG can be highly effective in this setting, only roughly half of epileptic discharges can be successfully recorded in affected patients, and healthy individuals may also exhibit false-positive results in this setting (11). As epileptic seizures can occur suddenly and are transient in nature, this can further complicate diagnostic efforts. As such, there is a clear need for the establishment of reliable, accurate, and specific approaches to diagnosing lTLE in order to guide patient care efforts.

The development of novel neuroimaging platforms holds great promise as a means of diagnosing lTLE and other neurological diseases. For example, resting state functional magnetic resonance imaging (rs-fMRI) is a non-invasive blood oxygen level-dependent neuroimaging strategy that can be used to directly visualize and assess functional connections among regions of the brain in a quantitative manner, allowing for the interrogation of neural network connections between the hemispheres of the brain (12). Several rs-fMRI studies to date have shown that TLE patients exhibit specific changes in brain network functionality, particularly in the default mode network (DMN), suggesting that these individuals may be at an elevated risk of experiencing cognitive decline (13, 14). Abnormal functional connectivity is thus likely to underlie declines in cognitive function and performance in individuals diagnosed with TLE, with several studies having explored this topic. Recently, voxel-mirrored homotopic connectivity (VMHC) was proposed as a conceptual approach to characterizing the synchronicity of spontaneous functional activity between geometrically consistent mirrored regions in the two cerebral hemispheres (15). VMHC values can be used to gain quantitative insight regarding functional connections based on time series correlations between mirrored voxels on either side of the brain. Abnormal VMHC values have been observed in the context of diseases including depression (16), schizophrenia (17), congenital amusia (18), diabetes mellitus (19), hyperthyroidism (20), and Parkinson’s disease (21). As such, measuring VMHC represents a sensitive strategy that can be leveraged to evaluate altered interhemispheric coordination in physiological and pathological contexts. To date, however, VMHC-based studies of lTLE patients have been limited. In one report, Yang et al. (22) determined that individuals diagnosed with idiopathic generalized epilepsy and generalized tonic-clonic seizures exhibited significant increases in VMHC values in the bilateral medial anterior curvature and anterior cingulate gyrus, while negative correlations were observed between illness duration and VMHC values in the bilateral cerebellum, thalamus, and orbital frontal cortex in these patients. In light of these prior observations, the present study was developed based on the hypothesis that lTLE patients may exhibit abnormal VMHC in the DMN, and that these altered VMHC values may be correlated with the course of TLE symptoms such that studying VMHC in these patients may offer insight into the pathophysiology of cognitive dysfunction in this patient population.

Artificial intelligence-based strategies have been used with increasing frequency in the context of diagnostic neuroimaging, with computer-aided SVM approaches being a subject of growing interest in this field that can aid in automating the diagnostic processing and identifying lesions. Owing to their high-resolution, rapidity, and non-invasive nature, neuroimaging techniques are commonly used to guide the diagnosis and evaluation of epilepsy patients. For example, abnormal degree centrality as a potential imaging biomarker for right temporal lobe epilepsy (10); decreased network homogeneity values in the right posterior cingulate cortex (PCC)/precuneus may be a potential neuroimaging marker for obsessive–compulsive disorder (23), and abnormal fractional amplitude of low-frequency fluctuation as a potential imaging biomarker for first-episode major depressive disorder (24).

Support vector machine (SVM) techniques enable the automated recognition of patterns within particular datasets (24), making them ideally suited to analyses of high-dimensional data types in which the number of potential features is greater than the number of samples available is common in the context of fMRI imaging. SVM approaches can identify an optimal separating hyperplane in high-dimensional space, with the closest instance of this hyperplane being referred to as a support vector. For fMRI analyses, SVM scales are overlaid onto the original functional space, with important scales then being plotted in different brain regions (25). Prior work has demonstrated the benefits of leveraging SVM techniques to transform high-dimensional neuroimaging data into information that can guide clinical decision-making (26), with SVM approaches having successfully used to differentiate between control individuals and persons diagnosed with major depressive disorder (27), schizophrenia (28), and bipolar disorder (29). No studies to date, however, have employed an SVM analytical approach to assess whether altered VMHC values can be used to differentiate between lTLE patients and controls.

Here, a combination of VMHC values and an SVM approach was utilized to assess resting-state functional connectivity between the two hemispheres of the brain and to examine how this relationship is linked to lTLE patient clinical characteristics. The overall goal of this approach was to establish the ability of altered VMHC values to facilitate the neuroimaging-based diagnosis of lTLE patients.

## Experimental procedures

### Participants

In total, 58 lTLE patients that had been diagnosed as per the criteria established by the International League Against Epilepsy (2017) were recruited for the present study from Tianyou Hospital affiliated with Wuhan University of Science and Technology. In parallel, 60 age-, sex-, and education level-matched healthy control (HC) participants were recruited. All lTLE patients met a minimum of two of the following criteria (30): a history of seizure-related symptoms consistent with the location of epileptic foci within the left temporal lobe; MRI or CT showed hippocampal sclerosis, atrophy, or temporal lobe lesions in the left temporal lobe, and interictal electroencephalographic traces revealing the presence of epileptic foci within the left temporal lobe. The exclusion criteria were as follows: age <14 years or age >60 years; patients who had a history of drug abuse or take drugs that could impair cognition, such as cannabis users and others; history of mental illness or systemic disease. exhibited a Mini-Mental State Examination (MMSE) score <24, presented with MRI findings consistent with the presence of other structural lesions in the brain such as tumors or vascular malformations, had suffered a traumatic brain injury, and exhibited contraindications that precluded MRI scanning. All participants provided written informed consent to participate. The Medical Ethics Committee of Tianyou Hospital affiliated with Wuhan University of Science and Technology approved this study, which was consistent with the Helsinki Declaration.

### Receive resting state functional magnetic resonance imaging

An Ingenia 3.0 T scanner (Philips, Amsterdam, Netherlands) equipped with a standard head coil was used to perform rs-fMRI scanning for all study participants. Scanning was conducted while participants remained still while lying down with their heads fixed in place with a belt. Foam padding and earplugs were used to mitigate scanner-related noise and head movements. Participants were directed to remain awake and not think about anything specific. rs-fMRI scans were performed with the following settings: repetition time = 2,000 ms, echo time = 25 ms, 36 axial slices, slice thickness = 3 mm, gap = 1 mm, 90° flip angle, field of view = 220 mm × 220 mm. The duration of rs-fMRI scanning for each participant was 8 min, with 240 volumes being obtained per participant.

### Data pre-processing

The MATLAB Data Processing Assistant for rs-fMRI (DPARSF) application was used to pre-process rs-fMRI data (31). The initial five time points for each participant were excluded from the analysis to mitigate the effects of initial signal instability and ambient scanner noise on the resultant data. Slice trimming was then conducted, after which the images were realigned to correct for any head movement. Participants were excluded from analysis if they exhibited >2 mm maximal displacement along the *x*, *y*, or *z* axes or >2° of maximal rotation. Data were then subjected to spatial registration in the standard Montreal Neurological Institute (MNI) space followed by resampling at 3 mm × 3 mm × 3 mm. The resultant images were then smoothed using a Gaussian kernel, linearly detrended, and subjected to bandpass filtering (0.01–0.08 Hz). Covariates such as head movement parameters, average whole-brain signals, white-matter signal, and signal derived from a defined ventricular region of interest were removed. Global signal was retained throughout rs-fMRI connectivity data processing.

### Voxel-mirrored homotopic connectivity analyses

The RSET toolkit^1^ was used to conduct VMHC analyses. Prior to these analyses, images were standardized to a symmetrical spatial template as follows: an average image for all participants was generated by averaging all normalized gray matter images; the established mean image was averaged with its bilateral mirror version to produce a symmetrical template mask to facilitate VMHC statistical analyses; and individual gray matter images were registered to this template, followed by non-linear transformation to yield functional images. Images were then smoothed using a 6 mm full-width at half-maximum isotropic Gaussian kernel. The time-series data for each voxel in the cerebral hemisphere were then extracted for each participant group following pretreatment and registration to the standard Montreal (MNI) space, after which Pearson correlation coefficients were calculated between individual voxels in symmetrical positions on either side of the brain to generate VMHC values. The resultant data were then converted *via* Fisher Z-transformation into a *Z*-value graph to facilitate subsequent statistical comparisons between groups. VMHC statistical analyses were performed using cerebral hemispheres in the symmetric template generated above. A previous study has described the details of VMHC acquisition (32).

### Statistical analyses

SPSS 22.0 was used to analyze all data. Results were reported as x ± s. Demographic and clinical data were compared between lTLE patients and HCs using independent sample *t*-tests, whereas gender ratios were compared between groups using chi-square tests. Whole-brain VMHC profiles for these two groups were subjected to voxel-based analyses of covariance to examine between-group differences, with results being Gaussian Random Field corrected at a threshold of *P* < 0.01.(voxel significance: *P* < 0.001; cluster significance: *P* < 0.01).

### Correlation analyses

Mean VMHC values from identified abnormal brain regions were extracted, and Pearson’s correlation analyses were employed to assess the relationship between these values and clinical parameters of interest.

### Classification analyses

The MATLAB LIBSVM package was used to implement an SVM analysis. The LIBSVM classifier was trained using providing examples of the form, where x represents the VMHC values of these abnormal clusters, and c is the class label (c = + 1 represent patients with lTLE while *c* = −1 for HCs). In order to evaluate the classification performance of unobserved data, the sample set of SVM was divided into training set and test set. We perform classification and feature selection by constructing random SVM cluster based on subjects’ brain fMRI data. The grid search method and default functional kernels of Gaussian radial basis were applied to optimize the parameters with the “leave-one-subject-out” method to acquire the optimal sensitivity and specificity. VMHC values extracted from the bilateral middle cingulum gyrus (MCG) and middle temporal gyrus (MTG) were assessed for their ability to differentiate between lTLE patients and HC individuals using this approach based the method.

## Results

In total, 58 patients with lTLE and 60 HCs were recruited for the present study. The clinical and demographic characteristics of these study participants are reported in Table 1. No significant differences in age, sex, disease course, or years of education were observed when comparing these groups.

**TABLE 1:** The *p*-value for gender distribution was obtained by the chi-square test.

Characteristics	Patients (*n* = 58)	HCs (*n* = 60)	*P*-value
Gender (male/female)	58 (37/21)	60 (31/29)	0.183
Age, years	28.97 ± 8.19	26.54 ± 4.96	0.052
Years of education, years	11.76 ± 1.90	12.67 ± 2.33	0.023
illness duration, years	5.76 ± 5.01		

The *p*-values were obtained by two sample *t*-tests.

HCs, healthy controls.

Compared with HCs, *P* < 0.01.

### Voxel-mirrored homotopic connectivity differences between groups

**FIGURE 1:**
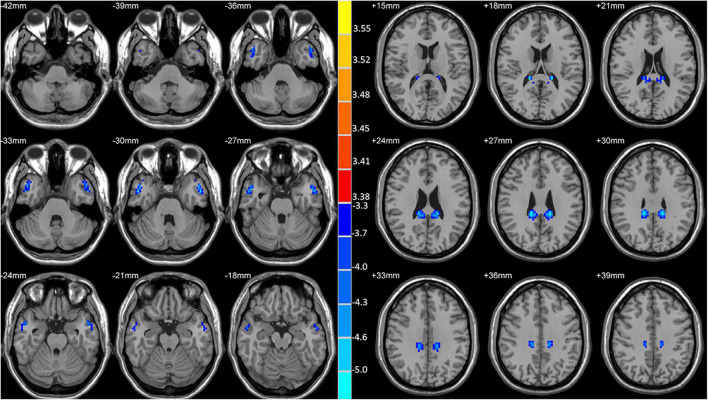
Statistical maps showing VMHC differences between the subject groups. Blue denotes lower VMHC, red denotes higher VMHC, and the color bar indicates the *T*-values from two-sample *t*-tests.

Significant reductions in VMHC values were observed in the bilateral MCG and MTG when comparing patients with lTLE to HC individuals (Figure 1 and Table 2). No analyzed brain regions exhibited increased VMHC values in individuals diagnosed with lTLE.

**TABLE 2:** Regions showing significant differences in VMHC between lTLE patients and HCs.

Cluster location	Peak (MNI)			Number of voxels	T-value
	X	Y	Z		
MTG	±51	0	−30	82	−4.9412
MCG	±15	−36	37	112	−5.3268

MNI, Montreal neurological institute; X, Y, Z, coordinate of primary peak locations in the MNI space. MTG, middle cingulum gyrus; MCG, middle cingulum gyrus.

### Support vector machine results

**FIGURE 2:**
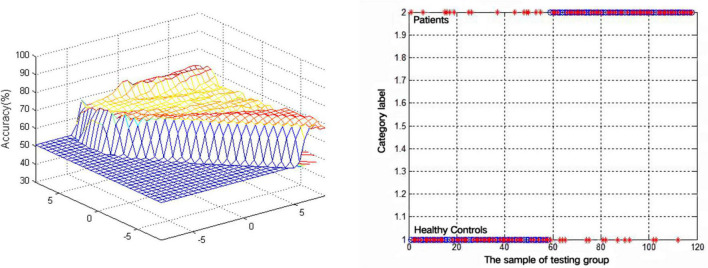
Visualization of classifications through support vector machine (SVM) using the decreased VMHC values in the MTG to discriminate lTLE patients from HCs. Left: SVM parameters result of 3D view. Right: Classified map of the VMHC values in the MTG.

An SVM approach was next separately used to analyze the observed VMHC reductions in the bilateral MCG and MTG in lTLE patients, revealing that the lower VMHC values in the MTG were associated with higher diagnostic accuracy (75.42%, 89/118) when differentiating between lTLE patients and HC individuals, with respective sensitivity and specificity values of 74.14% (43/58) and 76.67% (46/60) (Figure 2).

### Correlations between voxel-mirrored homotopic connectivity values and clinical parameters

Lastly, mean VMHC values were obtained for the bilateral MCG and MTG regions, with Pearson’s correlation analyses then being used to examine the relationship between these values and clinical parameters including age at seizure onset and disease duration. However, no significant correlations between VMHC values and these variables were detected in this patient cohort.

## Discussion

Here, rs-fMRI data were used to compare differences in interhemispheric VMHC values between lTLE patients and HC individuals. Relative to these controls, lTLE patients exhibited reduced VMHC values in the bilateral MTG and bilateral MCG. This is the first report to our knowledge to have employed an SVM approach to gauge the diagnostic utility of VMHC abnormalities in the bilateral MTG and MCG as an lTLE-related neuroimaging biomarker. This strategy ultimately revealed that reduced bilateral MTG VMHC values may offer significant value as a sensitive and specific biomarker capable of distinguishing between lTLE patients and HCs.

Prior work has shown that lTLE patients exhibit reduced VMHC values in the MTG, bilateral medial superior frontal gyrus, bilateral inferior parietal lobule, and supplementary motor area (33). Zhao et al. (34) observed significant reductions in the bilateral MTG connectivity in lTLE patients, in line with the VMHC results from the present study. The inferior temporal gyrus (ITG) and superior temporal gyrus (STG) are, respectively, located on the dorsal and ventral sides of the MTG. While once considered a structurally homogenous brain region (35), recent work has shown the MTG to play diverse roles in the context of social cognition, logical reasoning, memory, auditory processing, language, and emotion (9). One meta-analysis reported the MTG to be associated with the DMN and the semantic memory network (36). MTG impairment has been found to be associated with many different psychiatric and neurological disorders including autism spectrum disorder (37), major depressive disorder (38), bipolar disorder (39), TLE (40), and obsessive compulsive disorder (41). The MTG has also been identified as a promising target for surgical intervention in TLE patients *via* the *trans-*MTG approach (42). The MTG can be subdivided based on patterns of anatomical connectivity into the aMTG, mMTG, pMTG, and sMTG subregions. Of these, the aMTG is primarily connected to DMN-associated regions of the brain, indicating that it may be a critical component of the DMN (43). In contrast, the mMTG plays an essential role in the context of semantic memory (44), while the pMTG facilitates language processing, particularly in the context of repetition and reading (45), and the sMTG is linked to speech comprehension (46). Reductions in VMHC between the bilateral MTG has the potential to contribute to complex visual abnormalities, language disorders, memory impairment, and other cognitive deficits. In a study of patients with diabetes, researchers reported a positive correlation between MTG VMHC values and scores on the Montreal Cognitive Assessment Scale (19), with reductions in MTG VMHC values potentially explaining the cognitive impairment that these patients develop. As such, reduced MTG homotopy may similarly be linked to cognitive impairment in TLE patients.

The cingulate gyrus functions as an important mediator of learning and retention in addition to connecting the medial temporal lobe and the PCC (47, 48). The cingulum is a key marginal lobe component that connects different cerebral lobes together (48). The cingulate gyrus is broadly separated into four subregions based on structural characteristics and receptor distributions, including the anterior/PCC, the posterior splenic cortex, and the middle lingual cortex (49). Of these regions, the middle cingulate cortex has been linked to negative affect and cognitive control (50), primarily facilitating response selection based on the relevance of those potential responses to associated motivations (48). Several memory task-based analyses have shown the MCG in particular to be critically important in the context of working memory (51). Decreased PCC flexibility has been reported in individuals diagnosed with lTLE who experience memory impairment, particularly in the right hemisphere with a ∼22% reduction in connectional flexibility (52). These researchers were successfully able to utilize contralateral resting PCC flexibility as a biomarker to differentiate between individuals with and without memory impairment based on memory status, with an overall accuracy of 94% consistent with a link between PCC flexibility and memory function in lTLE patients. Other reports have identified significant shifts in normal resting-state activity in the cingulate gyrus in TLE patients, potentially accounting for the psychiatric symptoms, memory/learning deficits, and loss of consciousness that these patients experience. These results further highlight the potential for the impairment of the MCG to act as a critical node linked with lTLE-related cognitive dysfunction.

Here, reduced bilateral MTG and MCG VMHC values were evident in lTLE patients. The key regions of the DMN include the MTG and the PCC/precuneus (53), which coordinate processes associated with visuospatial functionality, self-reflection, and consciousness (54). Prior work has similarly confirmed that TLE patients exhibit reductions in functional connectivity in the DMN (40), and that repeated or prolonged epileptic discharges can impact this network. In one study, significant resting-state weakening was observed in several DMN-associated brain regions in individuals diagnosed with TLE (55). Consistently, medial TLE patients affected by hippocampal sclerosis were found to exhibit significant reductions in functional connection strength and structural connections between most regions of the brain in the DMN and other non-DMN regions of the brain (56). In line with these prior reports, the present study explored the link between the DMN and cognitive dysfunction in lTLE patients, with this relationship potentially linked to altered cognition and memory. In other reports, resting-state DMN activity has been shown to be significantly altered in individuals with TLE, potentially contributing to certain symptoms that these patients experience including psychiatric symptoms, memory or learning disorders, and loss of consciousness (57). The consistency between these previous reports and the present study further support a link between altered DMN activity and the pathophysiological development of TLE, with the reduced VMHC observed in lTLE patients in this study suggesting that reductions in DMN interhemispheric integration or coordination may contribute to the cognitive impairment experienced by patients with this disease. Roughly 40% of individuals diagnosed with epilepsy exhibit multiple forms of cognitive impairment (58), and prior work has revealed damage in several functional brain networks in patients with TLE including the alert network (59) and the executive network (40). These networks exhibit homotopy with respect to their structure and function, with the joint activity of both cerebral hemispheres being important for the maintenance of normal cognitive and emotional functionality. Altered information exchange or integration between these hemispheres can result in functional alterations such that the impaired homotopy observed in certain regions of the brain in individuals diagnosed with TLE may partially account for the functional deficits in these patients. Decreased VMHC values in the DMN thus offer neuroimaging-based support for prior evidence supporting a link between the pathogenesis of TLE and neurodegeneration.

The advent of increasingly advanced artificial intelligence strategies has been leveraged to guide neuroimaging-based computer-guided diagnostic efforts for patients with a range of neurological and pathological diseases. Novel MRI scanning and reconstructive strategies have been successfully leveraged to aid in diagnosing various diseases. Gao et al. (10), for example, found that a combination of elevated DC values in the left SFGdor and right SFGmed could be used as a neuroimaging biomarker for rTLE, with respective accuracy, sensitivity, and specificity values of 99.34, 100.00, and 98.55%. SVM strategies have been employed to aid in diagnosing psychiatric conditions such as schizophrenia (28) and major depression (24). Here, an SVM approach was used to assess abnormally altered VMHC values in the bilateral MTG and MCG, revealing that altered MTG VMHC values offered value as a biomarker capable of distinguishing between lTLE patients and HCs, with respective accuracy, sensitivity, and specificity values of 75.42, 74.14, and 76.67%. This study is the first to our knowledge to have explored the ability of altered MTG VMHC values to serve as an lTLE-related neuroimaging biomarker.

There are certain limitations to this analysis. For one, patients were treated for an extended period with AEDs, potentially altering rs-fMRI signals and associated study results. Indeed, as some AEDs have been shown to alter nervous system activation, it is not possible to exclude that AED treatment may have impacted the inter-group differences observed herein, underscoring the need for further research regarding the link between AED use and VMHC changes. Second, this was a cross-sectional study. Future longitudinal analyses are warranted to explore dynamic VMHC changes in particular regions of the brain. Lastly, this study was based on resting-state analyses, and further experiments combining both resting- and task-state fMRI have the potential to provide further insight regarding the magnitude of VMHC alterations in different regions of the brain under task conditions.

In summary, altered VHMC values in the bilateral MCG and MTG may correspond to altered resting-state activity in these areas in lTLE patients. Changes in VMHC values in the MTG may also offer great potential as a neuroimaging biomarker that can guide lTLE diagnosis.

## Data availability statement

The original contributions presented in this study are included in the article/supplementary material, further inquiries can be directed to the corresponding authors.

## Ethics statement

The studies involving human participants were reviewed and approved by the Ethics Committee of the Tianyou Hospital Affiliated to Wuhan University of Science and Technology. Written informed consent to participate in this study was provided by the participants’ legal guardian/next of kin.

## Author contributions

JW, RG, LC, and HR: conceptualization, project planning and methodology, and manuscript review and editing. JLW, SZ, and JL: data analysis and manuscript first draft. All authors contributed to the article and approved the submitted version.

## Footnotes

1
http://www.restfmri.net/forum/

## References

[1] Li DB Liu RS Wang X Xiong PA Ren HW Wei YF Abnormal ventral attention network homogeneity in patients with right temporal lobe epilepsy. *Eur Rev Med Pharmacol Sci.* (2021) 25:2031–8. 10.26355/eurrev_202102_25107 33660815

[2] Beghi E Giussani G Nichols E Abd-Allah F Abdela J Abdelalim A Global, regional, and national burden of epilepsy, 1990–2016: a systematic analysis for the global burden of disease study 2016. *Lancet Neurol.* (2019) 18:357–75. 10.1016/S1474-4422(18)30454-X 30773428 PMC6416168

[3] Gao Y Zheng J Li Y Guo D Wang M Cui X Abnormal default-mode network homogeneity in patients with temporal lobe epilepsy. *Medicine (Baltimore).* (2018) 97:e11239. 10.1097/MD.0000000000011239 29952987 PMC6039636

[4] Chen Z Brodie MJ Liew D Kwan P. Treatment outcomes in patients with newly diagnosed epilepsy treated with established and new antiepileptic drugs: a 30-year longitudinal cohort study. *JAMA Neurol.* (2018) 75:279–86. 10.1001/jamaneurol.2017.3949 29279892 PMC5885858

[5] Alqahtani F Imran I Pervaiz H Ashraf W Perveen N Rasool MF Non-pharmacological interventions for intractable epilepsy. *Saudi Pharm J.* (2020) 28:951–62. 10.1016/j.jsps.2020.06.016 32792840 PMC7414058

[6] Chang YA Kemmotsu N Leyden KM Kucukboyaci NE Iragui VJ Tecoma ES Multimodal imaging of language reorganization in patients with left temporal lobe epilepsy. *Brain Lang.* (2017) 170:82–92. 10.1016/j.bandl.2017.03.012 28432987 PMC5507363

[7] Bruder JC Wagner K Lachner-Piza D Klotz KA Schulze-Bonhage A Jacobs J. Mesial-temporal epileptic ripples correlate with verbal memory impairment. *Front Neurol.* (2022) 13:876024. 10.3389/fneur.2022.876024 35720106 PMC9204013

[8] Zhang Z Zhou X Liu J Qin L Ye W Zheng J. Aberrant executive control networks and default mode network in patients with right-sided temporal lobe epilepsy: a functional and effective connectivity study. *Int J Neurosci.* (2020) 130:683–93. 10.1080/00207454.2019.1702545 31851554

[9] Chang W Lv Z Pang X Nie L Zheng J. The local neural markers of MRI in patients with temporal lobe epilepsy presenting ictal panic: a resting resting-state postictal fMRI study. *Epilepsy Behav.* (2022) 129:108490. 10.1016/j.yebeh.2021.108490 35180570

[10] Gao Y Xiong Z Wang X Ren H Liu R Bai B Abnormal degree centrality as a potential imaging biomarker for right temporal lobe epilepsy: a resting-state functional magnetic resonance imaging study and support vector machine analysis. *Neuroscience.* (2022) 487:198–206. 10.1016/j.neuroscience.2022.02.004 35158018

[11] Liu G Xiao R Xu L Cai J. Minireview of epilepsy detection techniques based on electroencephalogram signals. *Front Syst Neurosci.* (2021) 15:685387. 10.3389/fnsys.2021.685387 34093143 PMC8173051

[12] Wu L Wang C Liu J Guo J Wei Y Wang K Voxel-mirrored homotopic connectivity associated with change of cognitive function in chronic pontine stroke. *Front Aging Neurosci.* (2021) 13:621767. 10.3389/fnagi.2021.621767 33679376 PMC7929989

[13] Gao Y Wang M Yu R Li Y Yang Y Cui X Abnormal default mode network homogeneity in treatment-naive patients with first-episode depression. *Front Psychiatry.* (2018) 9:697. 10.3389/fpsyt.2018.00697 30618871 PMC6305293

[14] Zhang Y Huang G Liu M Li M Wang Z Wang R Functional and structural connective disturbance of the primary and default network in patients with generalized tonic-clonic seizures. *Epilepsy Res.* (2021) 174:106595. 10.1016/j.eplepsyres.2021.106595 33993017

[15] Hu G Ge H Yang K Liu D Liu Y Jiang Z Altered static and dynamic voxel-mirrored homotopic connectivity in patients with frontal glioma. *Neuroscience.* (2022) 490:79–88. 10.1016/j.neuroscience.2022.03.006 35278629

[16] Zhang Y Mu Y Li X Sun C Ma X Li S Improved interhemispheric functional connectivity in postpartum depression disorder: associations with individual target-transcranial magnetic stimulation treatment effects. *Front Psychiatry.* (2022) 13:859453. 10.3389/fpsyt.2022.859453 35370853 PMC8964485

[17] Yang G Zhang S Zhou Y Song Y Hu W Peng Y Increased resting-state interhemispheric functional connectivity of striatum in first-episode drug-naive adolescent-onset schizophrenia. *Asian J Psychiatr.* (2022):103134. 10.1016/j.ajp.2022.103134 35551877

[18] Jin Z Huyang S Jiang L Yan Y Xu M Wang J Increased resting-state interhemispheric functional connectivity of posterior superior temporal gyrus and posterior cingulate cortex in congenital amusia. *Front Neurosci.* (2021) 15:653325. 10.3389/fnins.2021.653325 33994929 PMC8120159

[19] Zhang Y Wang J Wei P Zhang J Zhang G Pan C Interhemispheric resting-state functional connectivity abnormalities in type 2 diabetes patients. *Ann Palliat Med.* (2021) 10:8123–33. 10.21037/apm-21-1655 34353097

[20] Zhi M Hou Z Zhang Y Yue Y Li L Yuan Y. Cognitive Deficit-Related interhemispheric asynchrony within the medial hub of the default mode network aids in classifying the hyperthyroid patients. *Neural Plast.* (2018) 2018:9023604. 10.1155/2018/9023604 30532774 PMC6250039

[21] Gan C Wang L Ji M Ma K Sun H Zhang K Abnormal interhemispheric resting state functional connectivity in Parkinson’s disease patients with impulse control disorders. *NPJ Parkinsons Dis.* (2021) 7:60. 10.1038/s41531-021-00205-7 34272398 PMC8285494

[22] Yang T Ren J Li Q Li L Lei D Gong Q Increased interhemispheric resting-state in idiopathic generalized epilepsy with generalized tonic-clonic seizures: a resting-state fMRI study. *Epilepsy Res.* (2014) 108:1299–305. 10.1016/j.eplepsyres.2014.06.010 25043752

[23] Chen Y Ou Y Lv D Yang R Li S Jia C Altered network homogeneity of the default-mode network in drug-naive obsessive-compulsive disorder. *Prog Neuropsychopharmacol Biol Psychiatry.* (2019) 93:77–83. 10.1016/j.pnpbp.2019.03.008 30905622

[24] Gao Y Wang X Xiong Z Ren H Liu R Wei Y Abnormal fractional amplitude of low-frequency fluctuation as a potential imaging biomarker for first-episode major depressive disorder: a resting-state fMRI study and support vector machine analysis. *Front Neurol.* (2021) 12:751400. 10.3389/fneur.2021.751400 34912284 PMC8666416

[25] Qiu Y Yang M Li S Teng Z Jin K Wu C Altered fractional amplitude of low-frequency fluctuation in major depressive disorder and bipolar disorder. *Front Psychiatry.* (2021) 12:739210. 10.3389/fpsyt.2021.739210 34721109 PMC8548428

[26] Zhang H Li X Pang J Zhao X Cao S Wang X Predicting SSRI-resistance: clinical features and tagSNPs prediction models based on support vector machine. *Front Psychiatry.* (2020) 11:493. 10.3389/fpsyt.2020.00493 32581871 PMC7283444

[27] Yan M He Y Cui X Liu F Li H Huang R Disrupted regional homogeneity in melancholic and non-melancholic major depressive disorder at rest. *Front Psychiatry.* (2021) 12:618805. 10.3389/fpsyt.2021.618805 33679477 PMC7928375

[28] Jin K Xu D Shen Z Feng G Zhao Z Lu J Distinguishing hypochondriasis and schizophrenia using regional homogeneity: a resting-state fMRI study and support vector machine analysis. *Acta Neuropsychiatr.* (2021) 33:182–90. 10.1017/neu.2021.9 33818354

[29] Jan Z Ai-Ansari N Mousa O Abd-Alrazaq A Ahmed A Alam T The role of machine learning in diagnosing bipolar disorder: scoping review. *J Med Internet Res.* (2021) 23:e29749. 10.2196/29749 34806996 PMC8663682

[30] Manford M Fish DR Shorvon SD. An analysis of clinical seizure patterns and their localizing value in frontal and temporal lobe epilepsies. *Brain.* (1996) 119:17–40. 10.1093/brain/119.1.17 8624679

[31] Chao-Gan Y Yu-Feng Z. DPARSF: a MATLAB toolbox for “pipeline” data analysis of resting-state fMRI. *Front Syst Neurosci.* (2010) 4:13. 10.3389/fnsys.2010.00013 20577591 PMC2889691

[32] Zuo XN Kelly C Di Martino A Mennes M Margulies DS Bangaru S Growing together and growing apart: regional and sex differences in the lifespan developmental trajectories of functional homotopy. *J Neurosci.* (2010) 30:15034–43. 10.1523/JNEUROSCI.2612-10.2010 21068309 PMC2997358

[33] Liu HH Wang J Chen XM Li JP Ye W Zheng J. Interhemispheric functional and structural alterations and their relationships with alertness in unilateral temporal lobe epilepsy. *Eur Rev Med Pharmacol Sci.* (2016) 20:1526–36. 27160125

[34] Zhao X Kang H Zhou Z Hu Y Li J Li S Interhemispheric functional connectivity asymmetry is distinctly affected in left and right mesial temporal lobe epilepsy. *Brain Behav.* (2022) 12:e2484. 10.1002/brb3.2484 35166072 PMC8933759

[35] Briggs RG Tanglay O Dadario NB Young IM Fonseka RD Hormovas J The unique fiber anatomy of middle temporal gyrus default mode connectivity. *Oper Neurosurg (Hagerstown).* (2021) 21:E8–14. 10.1093/ons/opab109 33929019 PMC8203421

[36] Jeffrey R Binder RHDW Conant LL. Where is the semantic system? A critical review and meta-analysis of 120 functional neuroimaging studies. *Cereb Cortex.* (2009) 19:2767–96. 19329570 10.1093/cercor/bhp055 PMC2774390

[37] Ro A Ksab C Jma B Jt C Rh A Ty B Abnormal cortical activation during silent reading in adolescents with autism spectrum disorder. *Brain Dev.* (2019) 41:234–44. 10.1016/j.braindev.2018.10.013 30448302

[38] Cheng C Dong D Jiang Y Ming Q Zhong X Sun X State-related alterations of spontaneous neural activity in current and remitted depression revealed by resting-state fMRI. *Front Psychol.* (2019) 10:245. 10.3389/fpsyg.2019.00245 30804860 PMC6378291

[39] Hwang M Roh YS Talero J Cohen BM Baker JT Brady RO Auditory hallucinations across the psychosis spectrum: evidence of dysconnectivity involving cerebellar and temporal lobe regions. *Neuroimage Clin.* (2021) 32:102893. 10.1016/j.nicl.2021.102893 34911197 PMC8636859

[40] Gao YJ Wang X Xiong PG Ren HW Zhou SY Yan YG Abnormalities of the default-mode network homogeneity and executive dysfunction in people with first-episode, treatment-naive left temporal lobe epilepsy. *Eur Rev Med Pharmacol Sci.* (2021) 25:2039–49. 10.26355/eurrev_202102_25108 33660816

[41] Fan S Cath DC van den Heuvel OA van der Werf YD Schols C Veltman DJ Abnormalities in metabolite concentrations in tourette’s disorder and obsessive-compulsive disorder-A proton magnetic resonance spectroscopy study. *Psychoneuroendocrinology.* (2017) 77:211–7. 10.1016/j.psyneuen.2016.12.007 28104554

[42] Bozkurt B Da SCR Chaddad-Neto F Da CM Goiri MA Karadag A Transcortical selective amygdalohippocampectomy technique through the middle temporal gyrus revisited: an anatomical study laboratory investigation. *J Clin Neurosci.* (2016) 34:237–45. 10.1016/j.jocn.2016.05.035 27499121

[43] Fan L Wang J Zhang Y Han W Yu C Jiang T. Connectivity-based parcellation of the human temporal pole using diffusion tensor imaging. *Cereb Cortex.* (2014) 24:3365–78. 10.1093/cercor/bht196 23926116

[44] Cho S Metcalfe AW Young CB Ryali S Geary DC Menon V. Hippocampal-prefrontal engagement and dynamic causal interactions in the maturation of children’s fact retrieval. *J Cogn Neurosci.* (2012) 24:1849–66. 10.1162/jocn_a_00246 22621262 PMC3462165

[45] Saur D Kreher BW Schnell S Kummerer D Kellmeyer P Vry MS Ventral and dorsal pathways for language. *Proc Natl Acad Sci USA.* (2008) 105:18035–40. 10.1073/pnas.0805234105 19004769 PMC2584675

[46] Xu J Wang J Fan L Li H Zhang W Hu Q Tractography-based parcellation of the human middle temporal gyrus. *Sci Rep.* (2015) 5:18883. 10.1038/srep18883 26689815 PMC4686935

[47] Bubb EJ Metzler-Baddeley C Aggleton JP. The cingulum bundle: anatomy, function, and dysfunction. *Neurosci Biobehav Rev.* (2018) 92:104–27. 10.1016/j.neubiorev.2018.05.008 29753752 PMC6090091

[48] Maldonado IL Parente DMV Castro CT Herbet G Destrieux C. The human cingulum: from the limbic tract to the connectionist paradigm. *Neuropsychologia.* (2020) 144:107487. 10.1016/j.neuropsychologia.2020.107487 32470344

[49] Vogt BA. Pain and emotion interactions in subregions of the cingulate gyrus. *Nat Rev Neurosci.* (2005) 6:533–44. 10.1038/nrn1704 15995724 PMC2659949

[50] Tolomeo S Christmas D Jentzsch I Johnston B Sprengelmeyer R Matthews K A causal role for the anterior mid-cingulate cortex in negative affect and cognitive control. *Brain.* (2016) 139:1844–54. 10.1093/brain/aww069 27190027

[51] Heany SJ Phillips N Brooks S Fouche JP Myer L Zar H Neural correlates of maintenance working memory, as well as relevant structural qualities, are associated with earlier antiretroviral treatment initiation in vertically transmitted HIV. *J Neurovirol.* (2020) 26:60–9. 10.1007/s13365-019-00792-5 31482439

[52] Douw L Leveroni CL Tanaka N Emerton BC Cole AJ Reinsberger C Loss of resting-state posterior cingulate flexibility is associated with memory disturbance in left temporal lobe epilepsy. *PLoS One.* (2015) 10:e131209. 10.1371/journal.pone.0131209 26110431 PMC4481466

[53] Yeshurun Y Nguyen M Hasson U. The default mode network: where the idiosyncratic self meets the shared social world. *Nat Rev Neurosci.* (2021) 22:181–92. 10.1038/s41583-020-00420-w 33483717 PMC7959111

[54] Qin P Wu X Wu C Wu H Zhang J Huang Z Higher-order sensorimotor circuit of the brain’s global network supports human consciousness. *Neuroimage.* (2021) 231:117850. 10.1016/j.neuroimage.2021.117850 33582277 PMC9583596

[55] Laufs H Hamandi K Salek-Haddadi A Kleinschmidt AK Duncan JS Lemieux L. Temporal lobe interictal epileptic discharges affect cerebral activity in “default mode” brain regions. *Hum Brain Mapp.* (2007) 28:1023–32. 10.1002/hbm.20323 17133385 PMC2948427

[56] Zhang Z Lu G Zhong Y Tan Q Chen H Liao W FMRI study of mesial temporal lobe epilepsy using amplitude of low-frequency fluctuation analysis. *Hum Brain Mapp.* (2010) 31:1851–61. 10.1002/hbm.20982 20225278 PMC6870704

[57] Stretton J Pope RA Winston GP Sidhu MK Symms M Duncan JS Temporal lobe epilepsy and affective disorders: the role of the subgenual anterior cingulate cortex. *J Neurol Neurosurg Psychiatry.* (2015) 86:144–51. 24876189 10.1136/jnnp-2013-306966 PMC4316913

[58] Coimbra ER Rezek K Escorsi-Rosset S Landemberger MC Castro RM Valadao MN Cognitive performance of patients with mesial temporal lobe epilepsy is not associated with human prion protein gene variant allele at codons 129 and 171. *Epilepsy Behav.* (2006) 8:635–42. 10.1016/j.yebeh.2006.02.007 16580884

[59] Nie L Jiang Y Lv Z Pang X Liang X Chang W A study of brain functional network and alertness changes in temporal lobe epilepsy with and without focal to bilateral tonic-clonic seizures. *BMC Neurol.* (2022) 22:14. 10.1186/s12883-021-02525-w 34996377 PMC8740350

